# The impermanent effect of waste incineration on children’s development from 6 months to 8 years: A Taiwan Birth Cohort Study

**DOI:** 10.1038/s41598-020-60039-w

**Published:** 2020-02-21

**Authors:** For-Wey Lung, Bih-Ching Shu, Tung-Liang Chiang, Shio-Jean Lin

**Affiliations:** 1 Calo Psychiatric Center, Pingtung County, Taiwan; 2 Graduate Institute of Medical Science, National Defense Medical Center, Taipei, Taiwan; 3 Institute of Allied Health Sciences, Department of Nursing, College of Medicine, National Cheng Kung University, Tainan, Taiwan; 4 Institute of Health Policy and Management, College of Public Health, National Taiwan University, Taipei, Taiwan; 5 Genetic Counseling Center, Chi Mei Medical Center, Tainan, Taiwan

**Keywords:** Population screening, Risk factors

## Abstract

Incineration is a solution to waste problems; however, it has adverse effects on human health. Our study aimed to investigate the direct and indirect effects of living near an incinerator and breastfeeding on children’s development at 6, 18, 36 and 66 months, and 8 years of age. The Taiwan Birth Cohort Study dataset used included randomized community data on 19,519 children from 6 months to 8 years old. The Taiwan Birth Cohort Study Developmental Instrument was used to measure children’s development at different developmental ages. The results of our study showed that living within 3 km of an incinerator had a negative effect on children’s 6-month development, however the effect dissipated after 18 months. Having been breastfed and living in the city had a more persistent and pervasive positive effect on children’s development. Conversely, living in the city had an adverse effect on children’s social-communication and emotional development when they were 8 years old; possibly due to the Chinese cultural characteristic of collectivism. Further follow-up of the long-term interactive effects of proximity to an incinerator and breastfeeding on children’s development and health is needed.

## Introduction

The management of municipal and industrial waste is a growing problem. Compared with the more traditional method of landfill, incineration is favorable, for it can greatly reduce the volume of the waste before it is sent to landfill sites. However, because incinerators have been shown to have adverse effects on the environment and human health^1^, their use is controversial.

The effect of environmental exposure on human health starts as early as pre-pregnancy. Environmental exposure of both parents during pregnancy and development can lead to preterm delivery, congenital abnormalities and miscarriage^1^. Given that children are continuously undergoing neurological and physical changes, they are more vulnerable to the harmful effects of toxins^2^. A previous study on the effect of incinerator pollutants on children found an increase in behavioral problems associated with higher prenatal dioxin exposure^3^. Another study found that those with elevated levels of dioxins were more vulnerable to learning disability and attention deficit disorder^4^, showing that incinerators have a lasting effect on the development of children.

In addition to direct effects, children can also be exposed to the indirect effects of the oral intake of pollutants^5^. Although breastfeeding has generally been found to be beneficial to children’s cognitive development^6^, incinerator pollutants are fat-soluble and persistent; therefore, they can accumulate in human fat tissue and enter breast milk^7^. Lorber and Phillips found an accumulation of six times the dioxin dose in infants who had been breastfed for a year when compared with those who had never been breastfed^8^. In the Netherlands, Weisglas-Kuperus *et al*. found that exposure to background levels of dioxins and polychlorinated biphenyls (PCBs) through breastfeeding had a negative effect on the immune system^9^. Similarly, the Taiwan Birth Cohort Study (TBCS) found that 18-month-old children who lived near an incinerator and were breastfed had an increased risk of typical developmental delay, autism spectrum disorder, attention deficit hyperactivity disorder, and other socio-emotional disability disorders compared with those who did not live near incinerators^10^. However, no developmental differences were found in children in the same dataset when followed up to 5 years of age^11^.

Therefore, the aim of our study was to follow-up on the pathway relationship of living near an incinerator, and the possible indirect effect of breastfeeding on children’s development at 6, 18, 36 and 66 months, and 8 years of age using the TBCS dataset, which included a national household sample of 19,519 children and families in Taiwan.

## Results

### Demographic distribution

Children and families that participated in all stages of the study (6, 18, 36, 66 months and 8 years old) were included in the final dataset, resulting in a dataset of 19,519 families. The demographic distribution of the participating children and parents is shown in Table 1. Of the 19,519 children, 16,100 (82.5%) were breastfed, almost half lived in the city (9201; 47.1%), and 865 (4.4%) had an incinerator less than 3 km from their homes.

**Table 1:** Demographics of parents and children (N= 19,516).

Variable	*n* (%)
**Sex**	
Male	10,227 (52.4)
Female	9289 (47.6)
Lives in the city	9201 (47.1)
Lives <3 km from an incinerator	865 (4.4)
Breastfed	16,100 (82.5)
Low birthweight (<2500 g)	1320 (6.8)
Premature (<36 weeks)	1625 (8.3)
**Maternal level of education**	
Illiterate	16 (0.1)
Elementary school	745 (3.8)
High school	9908 (50.8)
University	8187 (42.0)
Graduate school	660 (3.4)
**Paternal level of education**	
Illiterate	3 (0.0)
Elementary school	271 (1.4)
High school	10,214 (52.3)
University	7569 (38.8)
Graduate school	1459 (7.5)
Taiwan Birth Cohort Study Developmental Instrument (range)	Mean (SD)
**6-month**	
Gross motor (9~27)	22.71 (3.26)
Fine motor (6~18)	16.25 (1.72)
Language (8~24)	20.95 (2.36)
Social (3~9)	5.88 (1.42)
**18-month**	
Gross motor (5~15)	13.88 (1.41)
Fine motor (3~9)	7.93 (1.22)
Language (4~12)	10.01 (2.13)
Social (5~15)	13.17 (1.73)
**36-month**	
Gross motor (6~18)	16.75 (1.69)
Fine motor (4~12)	10.03 (1.72)
Language (4~12)	11.83 (0.76)
Social (5~15)	13.75 (1.49)
**66-month**	
Gross motor (4~12)	11.51 (0.91)
Fine motor (4~12)	11.08 (1.09)
Language (4~12)	11.74 (0.76)
Social (4~12)	11.16 (1.08)
**8-years**	
Emotion (5~15)	14.15 (1.27)
Cognitive (5~15)	13.15 (1.68)
Social-communication (2~6)	5.63 (0.75)

### Logistic regression

Logistic regression was used to analyze the effects of living near an incinerator, having been breastfed, and living in the city on children’s gross motor, fine motor, language and social development at 6, 18, 36 and 66 months and 8 years old (Table 2). Living less than 3 km from an incinerator had a negative effect on children’s 6-month social development (β = −0.06, p = 0.013). Overall, having been breastfed and living in the city had a positive effect on the development of children. Breastfeeding had a positive effect on children’s fine motor and language development at 18 and 36 months (β = 0.09, p < 0.001; β = 0.07, p < 0.001; β = 0.06, p < 0.001; β = 0.05, p = 0.041), and on language and social development at 66 months (β = 0.07, p = 0.008; β = 0.06, p = 0.004). Breastfeeding had a negative effect on children’s fine motor development at 6 months (β = −0.03, p = 0.019), and on social development at 18 and 36 months (β = −0.03, p = 0.017; β = −0.04, p = 0.007). Living in the city had a more widespread and continuous positive effect on the development of children, including an effect on gross motor and social development at 6 months (β = 0.04, p < 0.001; β = 0.08, p < 0.001), fine motor development at 18 and 36 months (β = 0.11, p < 0.001; β = 0.09, p < 0.001), language and social development at 66 months (β = 0.14, p < 0.001; β = 0.10, p < 0.001), and cognitive development at 8 years old (β = 0.04, p = 0.008). However, living in the city had a negative effect on the fine motor and language development at 6 months (β = −0.04, p = 0.001; β = −0.02, p = 0.005), social development at 18 months (β = −0.04, p < 0.001), gross motor development at 36 months (β = −0.02, p = 0.028), fine motor development at 66 months (β = −0.13, p < 0.001) and social-communication development at 8 years old (β = −0.04, p = 0.008).

**Table 2:** Logistic regression analysis of incinerator, breastfeeding, and city on children’s development at 6, 18, 36 and 66 months and 8 years old.

Independent variable	Dependent variable	β	SE	p
Lives near incinerator	6 mo social development	−0.06	0.03	0.013
Breastfed	6 mo fine motor development	−0.03	0.01	0.019
	18 mo fine motor development	0.09	0.02	<0.001
	18 mo language development	0.07	0.01	<0.001
	18 mo social development	−0.03	0.01	0.017
	36 mo fine motor development	0.06	0.01	<0.001
	36 mo language development	0.05	0.03	0.041
	36 mo social development	−0.04	0.02	0.007
	66 mo language development	0.07	0.03	0.008
	66 mo social development	0.06	0.02	0.004
Lives in the city	6 mo gross motor development	0.04	0.01	<0.001
	6 mo fine motor development	−0.04	0.01	0.001
	6 mo language development	−0.02	0.01	0.005
	6 mo social development	0.08	0.01	<0.001
	18 mo fine motor development	0.11	0.01	<0.001
	18 mo social development	−0.04	0.01	<0.001
	36 mo gross motor development	−0.02	0.01	0.028
	36 mo fine motor development	0.09	0.01	<0.001
	66 mo fine motor development	−0.13	0.02	<0.001
	66 mo language development	0.14	0.02	<0.001
	66 mo social development	0.10	0.02	<0.001
	8 yr cognitive development	0.04	0.01	0.008
	8 yr social-communication development	−0.08	0.01	<0.001

### Structural equation modeling

**Figure 1:**
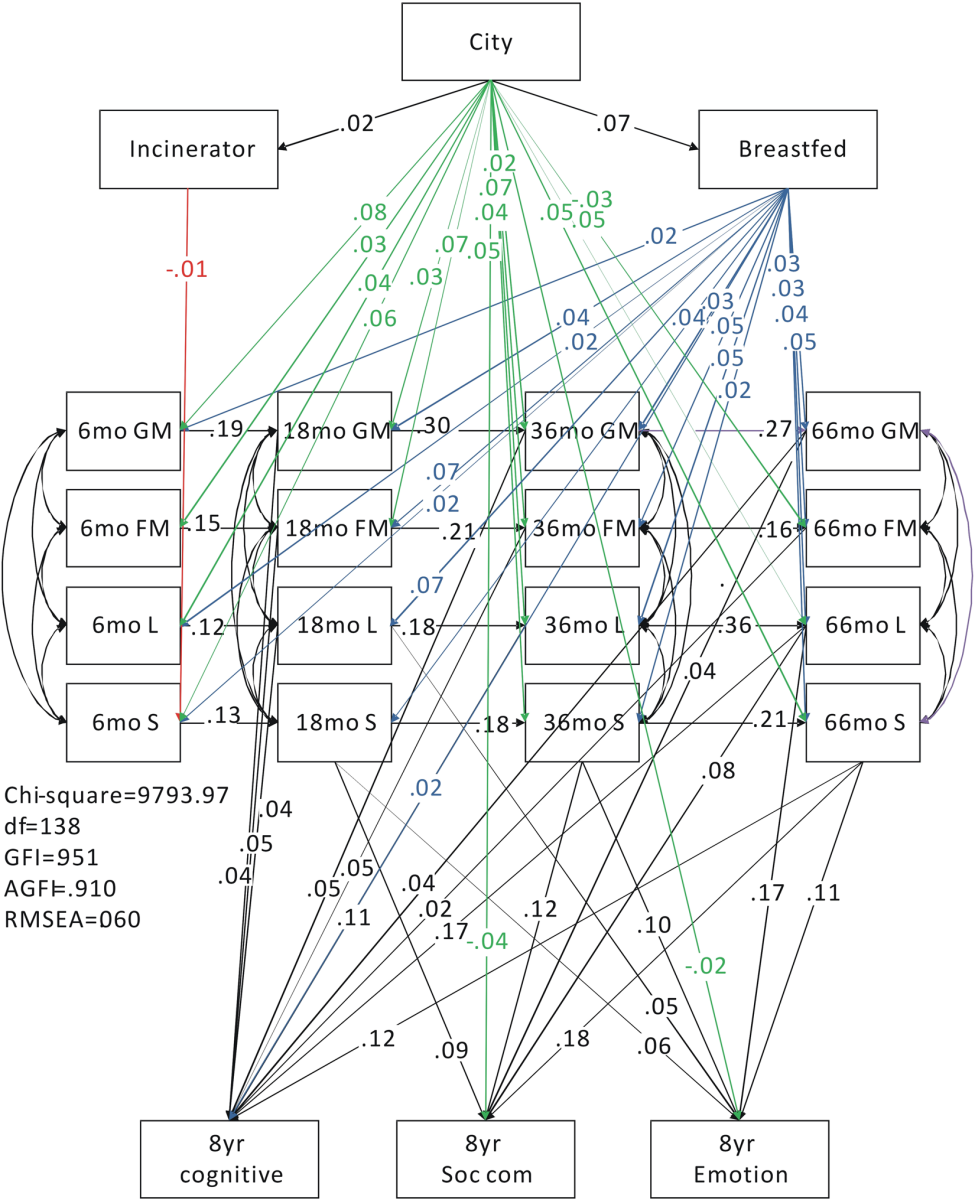
The pathway relationships among living near an incinerator, place of residence, and breastfeeding on children’s development from 6 months to 8 years. AGFI: adjusted goodness-of-fit; RMSEA: root mean square error of approximation; city (dummy variables: 1: city; 2: others).

Pathway analysis models were constructed to investigate the effects of living near an incinerator, having been breastfed, and living in the city on children’s gross motor, fine motor, language and social development at 6, 18, 36 and 66 months and 8 years old, including emotional, cognitive and social-communication development. The model resulted in a good fit, with an adjusted goodness-of-fit index (AGFI) of 0.910 (greater than 0.9) and a root mean square error of approximation (RMSEA) of 0.06 (less than 0.08), as shown in Fig. 1. The results showed that those living in the city also lived closer to incinerators, and these mothers were more likely to have breastfed their babies (β = 0.02, p = 0.002; β = 0.07, p < 0.001). The effect of living within 3  km of an incinerator was similar to that found in the regression analysis, with a negative effect on the social development of children at 6 months (β = −0.01, p = 0.038). Living in the city had a positive effect on children’s development from 6 to 66 months. Children who lived in the city had better gross motor, fine motor, language and social development at 6 months (β = 0.08, p < 0.001; β = 0.03, p < 0.001; β = 0.04, p < 0.001; β = 0.06, p < 0.001), gross motor and fine motor development at 18 months (β = 0.03, p < 0.001; β = 0.07, p < 0.001), gross motor, fine motor, language and social development at 36 months (β = 0.02, p = 0.009; β = 0.07, p < 0.001; β = 0.04, p < 0.001; β = 0.05, p < 0.001), and language and social development at 66 months (β = 0.05, p < 0.001; β = 0.05, p < 0.001). However, children living in the city had slower fine motor development at 66 months (β = −0.03, p < 0.001), and poorer social-communication and emotion development at 8 years old (β = −0.04, p < 0.001; β = −0.02, p = 0.002) when compared with children who did not live in the city. Finally, children that were breastfed showed a positive effect on their development from 6 to 66 months. Children that were breastfed had better gross motor, language and social development at 6 months (β = 0.02, p = 0.007; β = 0.02, p = 0.003; β = 0.02, p = 0.006), gross motor, fine motor, language and social development at 18 (β = 0.04, p < 0.001; β = 0.07, p < 0.001; β = 0.07, p < 0.001; β = 0.04, p < 0.001), 36 (β = 0.03, p < 0.001; β = 0.05, p < 0.001; β = 0.05, p < 0.001; β = 0.02, p = 0.002) and 66 months (β = 0.03, p < 0.001; β = 0.03, p < 0.001; β = 0.04, p < 0.001; β = 0.05, p < 0.001), and cognitive development at 8 years (β = 0.02, p < 0.001) compared with those that were not breastfed.

## Discussion

Using structural equation modeling, our birth cohort study from 6 months to 8 years of age found that living within 3 km of an incinerator had a temporary adverse effect on 6-month social development. No indirect effect of living near an incinerator was found through the mediating factor of breastfeeding on children’s development from 6 months to 8 years. Breastfeeding itself had a positive pervasive and persistent effect on children’s development from 6 months to 8 years old. In addition to having been breastfed, living in the city also had a pervasive and persistent positive effect on children’s development from 6 to 66 months. However, living in the city demonstrated an adverse effect on social-communication and emotional development when children were 8 years old.

Living near an incinerator had a temporary adverse effect on 6-month social development; however, the effect of living near an incinerator on children’s development dissipated after 6 months. This result is consistent with Lung and colleague’s latent growth curve study from 6 months to 5 years^11^. In addition, analysis of the TBCS dataset showed that living near an incinerator had an adverse effect on children’s initial development at 6 months; however, these children caught up in their development^11^. Conversely, the same dataset was used in a cross-sectional study to investigate the effect of proximity to an incinerator on children’s development at 6 and 18 months and 3 years, and found that proximity to an incinerator had an adverse effect on children’s development at 6 months and 3 years old^10^. These differences may be due to the different methods of analysis used. Our study combined the advantages of the previous two studies, including different dimensions of children’s development from 6 months to 8 years old, and combined the results at different stages of development in children followed-up to the age of 8 years, finding an impermanent effect of proximity to an incinerator on children’s development at 6 months.

Our study did not find an indirect effect on children’s development of living near an incinerator through the mediating factor of breastfeeding. Currently, the greatest source of PCB exposure in human is from food, since PCBs accumulate in food chains and are found in animal tissues^12^. Animal studies have found PCB exposure to alter brain capillary endothelium and connectivity in critical regions in the brain^13,14^. Regarding the influence of toxins in breastmilk, a study in Spain showed, although PCDD/Fs were found in breast milk from women living in the vicinity of a hazardous waste, however, it was mainly influenced by the mothers’ dietary intake of fish, meat, oils and fats^15^. Previous studies that reported an indirect effect of an incinerator through the mediating factor of breastfeeding mainly investigated the development of children’s mental and behavioral health, including background levels of dioxins and PCBs, finding that breastfeeding had a negative effect on the immune system and behavior^3^. Children living near an incinerator who were breastfed had an increased risk of typical developmental delay, autism spectrum disorder, attention deficit hyperactivity disorder, and other socio-emotional disability disorders when compared with those who did not live near incinerators^10^. These differences among studies could be due to the differences in the measurement systems used. Our study used a broad screening instrument for child development, and not the diagnostically oriented screening instrument used in previous studies^16^. Previous studies have proposed that the beneficial nutrients in breast milk counteract the effects of the dioxins in children’s cognitive performance^17,18^. This could explain for effect of incinerator and breastfeeding at when the children were younger in the Taiwan Birth Cohort Study^10^, and no differences found in this follow-up study.

Although no indirect effect on children’s development of living near an incinerator was found through the mediating factor of breastfeeding, breastfeeding itself had a pervasive and persistent positive direct effect on children’s development from 6 months to 8 years old. Previous studies have generally found breastfeeding to have a beneficial effect on cognitive development compared to only formula feeding infants^6^, for human breast milk provides essential fatty acids, including docosahexaenoic acid and arachidonic acid, that are important for central nervous system development^17^. Furthermore, children who were breastfed are shown to have greater grey matter than non-breastfed children^19^. The combination of nutritional and development factors of breastfeeding have significant, long-lasting consequences^20^. As shown in the results of our study with breastfeeding not only having impact on children’s cognitive develop, but also motor, language, and social development, up to the age of eight.

In addition to having been breastfed, living in the city also had positive pervasive and persistent effect on children’s development from 6 to 66 months. Conversely, living in the city had an adverse effect on children’s social-communication and emotional development at 8 years old. This is in line with the results of previous studies that found that children growing up in the city showed better development than those in rural areas^10,11^, reflecting differences in the socioeconomic neighborhood and urban–rural inequality. However, our follow-up to the age of 8 years found that living in the city becomes an adverse effect on children’s social-communication and emotional development. From a social construction perspective, communities connect us to a place where neighbors provide mutual support^21^. Communities also teach us to live with people different from us, so they teach us tolerance, co-existence, and mutual respect, whereas networks are based on choice^21^. However, the increasing complexity and mobility in urban environments makes it more difficult to sustain social and communal links. The urban disparity in emotional and social-communication development was not found at earlier stages of development, possibly because, after the age of 6 years, children enter schools and encounter more people and observe a wider range of emotions^22^. Parents introduce cultural and subcultural rules on emotions to their children. Asians live in a collectivistic context that emphasizes social harmony and achievement and honors the group/family; they are likely to exert more restraint in emotional expression than people living in individualistic cultures^23^. Furthermore, in a vertical hierarchical family structure, parents are encouraged to be strict and express less emotion, and children are encouraged to be obedient^24^. These restraints may be even more emphasized in urban areas.

A limitation of our study was that the possibility of toxin exposure was determined by the variable of whether the family reported to live within 3 km of a waste incinerator; therefore, no direct estimation of the exposure of the children to specific toxins, including measurements on dietary components or the environment, was made. However, municipal solid waste incinerators have been shown to be an important source of dioxin-like compounds (PCBs and polybrominated dibenzo-p-dioxins and dibenzofurans) in ambient air samples in Taiwan^25^.

In conclusion, our randomized household countrywide large birth cohort sample from 6 months to 8 years old found that living near a waste incinerator had an impermanent adverse effect on children’s development at 6 months of age. Having been breastfed and living in the city had a more persistent and pervasive effect on children’s development. Having been breastfed had a beneficial effect on children’s overall development and cognitive development up to the age of 8 years. Living in the city had a beneficial effect up to 5.5 years, but this turned into an adverse effect on children’s emotional and social-communication development at the age of 8 years. The long-term interactive effects of specific toxins and breastfeeding on children’s long-term development and health require investigation.

## Methods

### Participants

The TBCS 6-, 18-, 36-, 66-month- and 8-year-old datasets were used for this study. Through the method of national household probability sampling, the TBCS aimed to build a sample that would be representative of the children in Taiwan^26^. All babies born between October 2003 and January 2004 in Taiwan were eligible for inclusion in the TBCS, with no exclusion criteria^26^. A two-stage stratified random sampling method was used. In the first stage, the primary sampling unit was cities and towns; 85 of 369 townships were selected by systematic random sampling and then grouped into 12 strata based on four levels of urbanization and three levels of total fertility rate. In the second stage, newborns were proportionally selected from the 85 cities and towns according to the rate of birth. The final sample of 21,248 families (11.7% selection rate) was included at the first stage of data collection, when the children were 6 months old^26^. At 18 months, 20,172 families agreed to be followed up (95%), 19,910 (94%) families agreed to be followed up at 36 months, 19,721 (93%) at 66 months and, finally, 19,519 (92%) when the children were 8 years old. The TBCS protocol was approved by the institutional review board of Taipei City Hospital, Songde Branch and is in accordance with the Helsinki Declaration; after detailed explanation of the study, informed consent was obtained from the parents of all participants at each stage of the study.

### Materials

All information collected involved parental self-report. Information on exposure to the presence of an incinerator was collected by asking the parents whether there were incinerators within 3 km of their place of residence. Children’s development at 6, 18, 36 and 66 months and 8 years of age was measured using the Taiwan Birth Cohort Study – Developmental Instrument (TBCS-DI).

### TBCS-DI

The TBCS-DI is a short, culturally sensitive, parental-report developmental instrument that measures children’s gross motor, fine motor, language and communication (language), and social and emotional (social) developmental dimensions at 6, 18, 36 and 66 months; the 8-year-old scale measures children’s emotional, cognitive and social-communication development. In the 6-month scale, the TBCS-DI includes 26 items: 17 in the 18-month scale, 19 in the 36-month scale, 16 in the 66-month scale, and 12 in the 8-year-old scale. The 6-, 18-, 36-, 66-month and 8-year-old scales all showed good construct, predictive and content validity^27–29^. Parental response used a 3-point Likert scale for all scales, with higher scores implying better development.

### Data analysis

The demographic distribution of the children and parents was analyzed using SPSS for Windows 20.0 (Chicago, IL, USA). In addition, logistic regression was used to analyze whether an incinerator, location of residence and having been breastfed had an effect on children’s development from 6 months to 8 years old. Missing data were replaced using Bayesian analysis, an approach that produces a maximum likelihood estimate using all the available information. The combined use of Bayesian and pathway analysis to fill in missing data is suggested to be ideal for longitudinal studies of child development^26^.

Structural equation modeling (SEM) was used to analyze the effect of place of residence (in city or country), living near an incinerator and breastfeeding on the development of children from 6 months to 8 years. SEM presents a pathway relationship between all interested variables in a graphical form, which is helpful in investigations with many variables of interest, direct and indirect mediating effects (such as the relationship among living near an incinerator, breastfeeding, and children’s development in our study), and controlling for confounding factors (such as living in the city and living near an incinerator)^26^. SEM uses the χ^2^ distribution to test the overall fit of the data. An AGFI greater than 0.09 and an RMSEA less than 0.08 indicate that the model describes the observed data adequately. The pathway relationships among the investigated variables are represented by β values of regression or path coefficients. However, only parsimonious models are presented, which means that only statistically significant pathways (*p* values less than 0.05) are presented. Both the Bayesian analysis and pathway analysis were carried out using the AMOS 7.0 statistical software package (SPSS).

## Data Availability

The datasets underlying the results presented in the study are available from Bureau of Health Promotion (Taiwan) at http://www.bhp.doh.gov.tw/BHPnet/English/index.aspx.
